# Prediction for Mitosis-Karyorrhexis Index Status of Pediatric Neuroblastoma via Machine Learning Based ^18^F-FDG PET/CT Radiomics

**DOI:** 10.3390/diagnostics12020262

**Published:** 2022-01-20

**Authors:** Lijuan Feng, Luodan Qian, Shen Yang, Qinghua Ren, Shuxin Zhang, Hong Qin, Wei Wang, Chao Wang, Hui Zhang, Jigang Yang

**Affiliations:** 1 Department of Nuclear Medicine, Beijing Friendship Hospital, Capital Medical University, Beijing 100050, China; fenglijuan@mail.ccmu.edu.cn (L.F.); qianluodan1994@163.com (L.Q.); sunny_1869@126.com (S.Z.); 18611245486@163.com (W.W.); 2 Department of Surgical Oncology, Beijing Children’s Hospital, Capital Medical University, National Center for Children’s Health, Beijing 100045, China; nmgyangshen@126.com (S.Y.); 13263371301@163.com (Q.R.); qinhong999999@163.com (H.Q.); 3 Sinounion Medical Technology (Beijing) Co., Ltd., Beijing 100192, China; chao.wang@sinounion.com; 4 Department of Biomedical Engineering, School of Medicine, Tsinghua University, Beijing 100084, China; hzhang@tsinghua.edu.cn

**Keywords:** neuroblastoma, mitosis-karyorrhexis index, PET/CT, machine learning, nomogram

## Abstract

Accurate differentiation of intermediate/high mitosis-karyorrhexis index (MKI) from low MKI is vital for the further management of neuroblastoma. The purpose of this research was to investigate the efficacy of ^18^F-FDG PET/CT–based radiomics features for the prediction of MKI status of pediatric neuroblastoma via machine learning. A total of 102 pediatric neuroblastoma patients were retrospectively enrolled and divided into training (68 patients) and validation sets (34 patients) in a 2:1 ratio. Clinical characteristics and radiomics features were extracted by XGBoost algorithm and were used to establish radiomics and clinical models for MKI status prediction. A combined model was developed, encompassing clinical characteristics and radiomics features and presented as a radiomics nomogram. The predictive performance of the models was evaluated by AUC and decision curve analysis. The radiomics model yielded AUC of 0.982 (95% CI: 0.916, 0.999) and 0.955 (95% CI: 0.823, 0.997) in the training and validation sets, respectively. The clinical model yielded AUC of 0.746 and 0.670 in the training and validation sets, respectively. The combined model demonstrated AUC of 0.988 (95% CI: 0.924, 1.000) and 0.951 (95% CI: 0.818, 0.996) in the training and validation sets, respectively. The radiomics features could non-invasively predict MKI status of pediatric neuroblastoma with high accuracy.

## 1. Introduction

Neuroblastoma, the most common malignancy in infancy, profoundly contributes to childhood cancer deaths. A heterogeneous tumor has clinical outcomes ranging from spontaneous regression to extensive systemic metastasis [1]. Clinically, neuroblastoma progression is associated with local and/or distant metastasis and frequent relapses, with a rapidly decreasing timeline. For high-risk neuroblastoma children, the long-term survival rate is less than 40% regardless of intensive treatment [2]. Therefore, risk stratification is critical for selecting the best treatment for individuals in the era of precision medicine. According to Children’s Oncology Group (COG), independent prognostic indexes included age, histologic category, mitosis-karyorrhexis index (MKI) status, and grade. The International Neuroblastoma Pathology Classification (INPC) is based on age at diagnosis, differentiation grade of the neuroblasts, MKI, and the presence or absence of Schwannian stromal development [3,4]. Using the INPC system, neuroblastoma INPC classification is a strong prognostic index: favorable histology neuroblastoma has an overall survival of 84%, while 45% for unfavorable histology neuroblastoma [5]. The MKI refers to the total number of cells undergoing karyorrhexis or in mitosis, based on the assessment of a minimum of 5000 tumor cells. MKI results are then stratified as low (<100/5000 cells or <2%), intermediate (100 to 200/5000 cells or 2% to 4%), or high (>200/5000 cells or >4%). Revised Neuroblastoma Risk Classification System (NRCS): a report from the COG suggested that 5-year event-free survival of patients with intermediate or high MKI was higher than that of low MKI (78.8% vs 62.4%), and 5-year overall survival of low and intermediate/high MKI group had significant difference (89.9% vs 65.6%) [6]. Analysis of morphological parameters demonstrated that the MKI strongly correlated with overall survival [7]. Therefore, accurate differentiation of patients with intermediate/high MKI from low MKI is vital for further management.

Traditionally, the assessment of the MKI is based on a manual count of sufficient microscopic fields to include a minimum of 5000 cells. Depending on the pathologist, the count may be accomplished more or less strictly and is estimated rather than accurately calculated. For tumors that are highly mitotic, or at the opposite end of the range, MKI can be reliably estimated as high or low, respectively. For tumors that are closer to the cutoff values between intermediate and high or low and intermediate, or inconsistent from field to field, a more careful evaluation of MKI is essential [5]. Despite the progress in computer-assisted technology and image analysis, accurate evaluation of MKI status still faced some challenges. Meanwhile, as an invasive approach, the traditional biopsy may result in various complications [8], therefore, another non-invasive approach is needed to efficiently describe the status of MKI.

Radiomics analysis using multiparametric imaging can be utilized to produce high-throughput computation feature extraction, including tumor feature extraction, size, shape, feature intensity, which can subsequently be investigated to build radiomics models that can predict tumor pathology and prognosis. One of the most obvious values of radiomics study was to optimize patient-specific therapy paradigms [9]. The application of ^18^F-FDG PET/CT in neuroblastoma has been reported previously and has been confirmed its value in staging and prognosis prediction [10,11,12]. Radiomics analysis of ^18^F-FDG PET/CT can predict the status of TERTp-mutation status of high-grade gliomas [13], EGFR mutation in lung adenocarcinoma [14], hormone receptor distribution, proliferation rate, lymph node and distant metastasis of breast carcinoma [15]. The application of machine learning methodologies on histopathological images is a blossoming field with significant potential for clinical impact [16]. There have been no studies to date, however, which utilize radiomics based on ^18^F-FDG PET/CT to predict the MKI status in pediatric neuroblastoma. Therefore, the purpose of the present research was to investigate the efficacy of ^18^F-FDG PET/CT–based radiomics features for the prediction of MKI status of pediatric neuroblastoma via machine learning.

## 2. Materials and Methods

### 2.1. Patients

All of the included neuroblastoma patients underwent pre-therapy ^18^F-FDG PET/CT scans between March 2018 and November 2019 in our department. The inclusion criteria were as follows: (1) neuroblastoma confirmed by pathology; (2) age ≤ 18 years old at the time of diagnosis; (3) available PET/CT scan data; (4) available clinical information including demographic and clinical characteristics, routine lab indexes of neuroblastoma; (5) no tumor-related treatment before PET/CT; (6) available MKI results. The exclusion criteria included: (1) patients accepted chemotherapy before ^18^F-FDG PET/CT scan; (2) patients underwent ^18^F-FDG PET/CT after primary tumor excision.

According to the inclusion and exclusion criteria, 102 neuroblastoma patients were included for the MKI status prediction study (47 males and 55 females, mean age: 33.5 months, range: 17.0–52.3 months, 58 patients with low MKI, and 44 with intermediate/high MKI). All of the enrolled cases were randomly divided into a training set and a validation set according to the ratio of 2:1 (68 cases for training, 34 cases for validation). This retrospective study was performed according to the requirements of the Declaration of Helsinki and approved by the Institutional Review Board of Beijing Friendship Hospital, Capital Medical University (Ref. No. 2020-P2-091-02). Moreover, the requirement of written informed consent was waived.

### 2.2. Evaluation of the MKI

An MKI evaluation was performed on well-spread H&E-stained smears and their corresponding cell blocks. At least 5000 cells were assessed with a 40× objective in nonoverlapping areas, and the number of mitoses and karyorrhectic nuclei in the appointed areas was analyzed by two pathologists. Caution should be taken to avoid counting apoptotic nuclei. The final MKI results were expressed as a percentage, including low (<100/5000 cells or <2%), intermediate (100 to 200/5000 cells or 2% to 4%), or high (>200/5000 cells or >4%) [17].

### 2.3. Image Acquisition

All of the patients underwent PET/CT (Biograph mCT-64 PET/CT; Siemens, Knoxville, TN, USA) examinations following European Association of Nuclear Medicine guidelines for tumor imaging [18,19]. They were instructed to fast for at least 6 h and decrease intense exercises for at least 24 h, and 0.10–0.15MBq/kg of ^18^F-FDG was intravenously injected 40–60 min before the PET/CT scan. A low-dose CT scan (CT scanning characteristics: tube voltage 120 keV, resolution 0.586 × 0.586 mm, thickness 2 mm, matrix size 512 × 512) for viewing anatomic reference and attenuation correction was performed firstly, followed by PET scan. PET scan was performed with 3-dimension image mode and 2 min per bed position immediately after CT. The ordered subsets-expectation maximization algorithm in a time-of-flight based iterative reconstruction method was used for PET images reconstruction. All corrections, including detector efficiency, normalization, dead time, random counts, scatter, attenuation, were applied during reconstruction. A Gaussian smooth filter of 5 mm in full width at half-maximum was applied to the PET image.

### 2.4. Region of Interest Segmentation and Radiomics Features Extraction

**Figure 1:**
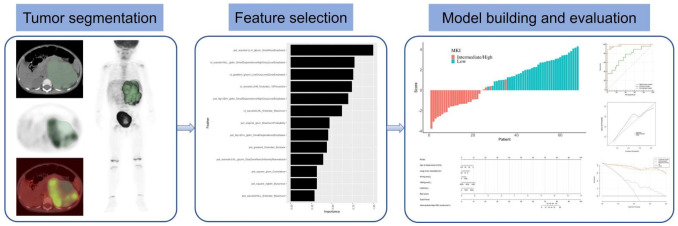
Workflow of the steps in our study.

The regions of interest (ROI) segmentation of the primary tumor were manually drawn by 3D Slicer (version 4.10.1). With PET images as a reference, ROIs were delineated along the edge of neuroblastoma lesions on CT images, including metastatic lesions with the unclear demarcation between the primary lesion and its surrounding metastatic lesions. The ROIs of all 102 patients were drawn by 2 different nuclear medicine physicians. Our study flow diagram is shown in Figure 1.

Radiomics features were extracted from both masked CT and PET images using Pyradiomics in Python (version 3.7.0). PET and CT images were discretized by equal width bins with standard uptake values of 0.3 and 25 CT values (Hu), respectively.

### 2.5. Machine Learning and Radiomics Features Selection

In this research, an extreme Gradient Boosting (XGBoost) algorithm was used to construct a robust machine learning based classification. The classifier was built using XGBoost (version 0.81) in Python. XGBoost algorithm is a scalable end-to-end tree boosting method that is widely applied by data researchers to acquire state-of-the-art results on many machine learning difficulties [20]. XGBoost belongs to assembly algorithms that form and combine a set of individually weak classifiers to yield a robust estimator. Matching with the XGBoost algorithm, a model-based features selection method was applied for tree learning algorithms. The features were ordered based on the importance across all of the decision trees within the model. The importance is evaluated for a single decision tree by the amount that each attribute split point enhances the performance, weighted by the number of observations [21]. The average importance of the subsets, which split from the training set randomly, was calculated as the selected reference. All of the features that contributed to the classification were selected to build the model. The radiomics features of each case were acquired from the score of the machine learning classification algorithm.

### 2.6. Model Construction and Evaluating Performance of the Models

A radiomics score (Rad-score) was counted for each patient from a linear combination of selected and weighted features by their correspondent coefficients, and the radiomics model was constructed by logistic regression based on Rad-score. XGBoost algorithm was also used to screen clinical characteristics for use in building clinical model. Finally, radiomics features and clinical characteristics were fitted to build the combined model and presented as a radiomics nomogram. The performance of each model was evaluated by the area under the receiver operator characteristic (ROC) curve (AUC). The calibration of the combined model was evaluated with calibration curves. Decision curve analysis (DCA) was used to estimate the clinical utility of the combined model, radiomics model, and clinical model in the training set.

### 2.7. Statistical Analysis

IBM SPSS Statistics (version 26.0) was used for statistical analysis in this study. Categorical variables are expressed as frequencies and percentages; continuous variables are expressed as median with interquartile range. The differences of patients’ characteristics between the training and validation set as well as between the low and intermediate/high MKI group were compared using two independent samples t-test or Mann Whitney U test. The Delong test was performed for evaluating differences in AUCs in various models. A 2-sided *p* < 0.05 indicated statistical significance.

## 3. Results

### 3.1. Patient Characteristics

The clinical characteristics of the training and validation sets are summarized in Table 1. No significant differences emerged in all of these clinical characteristics between training and validation sets. All of these clinical characteristics were compared between the low and intermediate/high groups in training and validation sets, respectively. Furthermore, there were no differences between the low and intermediate/high MKI groups in the training and validation sets (Table 2).

### 3.2. Radiomics Features Selection and Radiomics Model Construction

**Figure 2:**
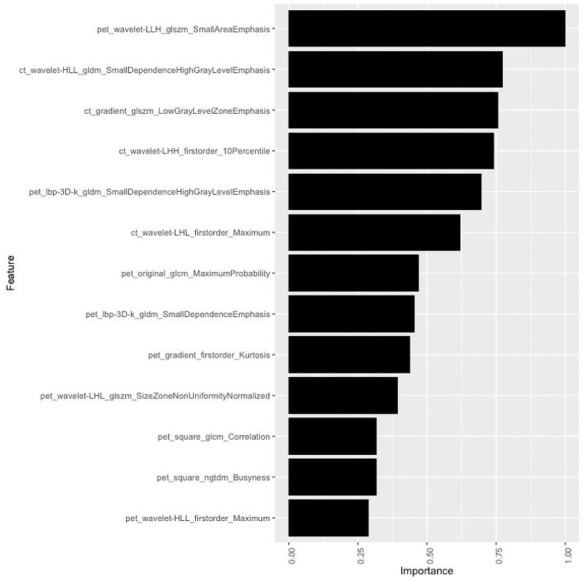
Radiomics features identified as important for the performance of XGboost.

**Figure 3:**
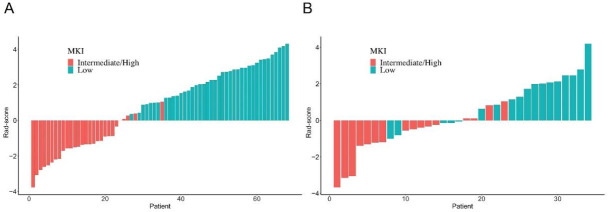
Rad-score of each patient. (**A**) Training set; (**B**) Validation set.

A total of 3384 radiomics features were extracted based on PET/CT images for each patient. Then XGBoost algorithm was conducted to identify the final 13 optimal features, and the coefficients of the corresponding features were calculated (Figure 2). Finally, the Rad-score (Figure 3) was calculated to build the radiomics model.

### 3.3. Clinical Model and Radiomics Nomogram Construction

**Figure 4:**
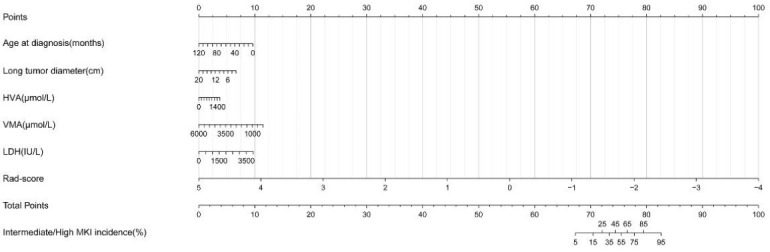
Radiomics nomogram of logistics regression for the combined model.

The XGBoost algorithm was used to finalize the five best clinical characteristics (age at diagnosis, serum lactate dehydrogenase (LDH), urine homovanillic acid (HVA) and vanillylmandelic acid (VMA), and long tumor diameter) for building the clinical model. The above five clinical characteristics and Rad-score were utilized for combined model construction, which was visualized by radiomics nomogram. And the radiomics nomogram (Figure 4) was created based on the training set.

### 3.4. Performance of Prediction Models

**Figure 5:**
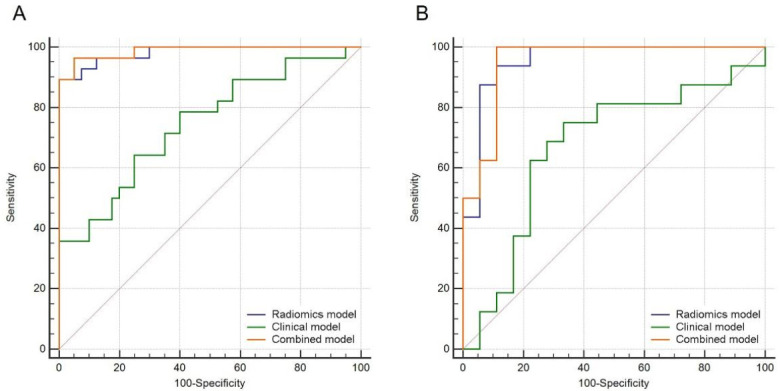
ROC curves for the combined model, clinical model and radiomics model in the (**A**) training and (**B**)validation sets.

The AUC of different models is presented in Table 3. Figure 5 shows the ROC curves of all of the models in both training and validation sets. The performance of the radiomics model (AUC, training set = 0.982, validation set = 0.955) is similar to the combined model (AUC, training set = 0.988, validation set = 0.951). Both the radiomics and combined models had better predictive performance for MKI status than the clinical model (AUC, training set = 0.746, validation set = 0.670).

**Figure 6:**
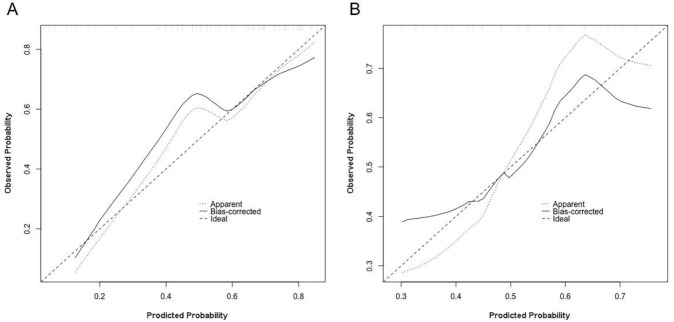
Calibration curves of the combined model in the (**A**) training and (**B**) validation sets.

**Figure 7:**
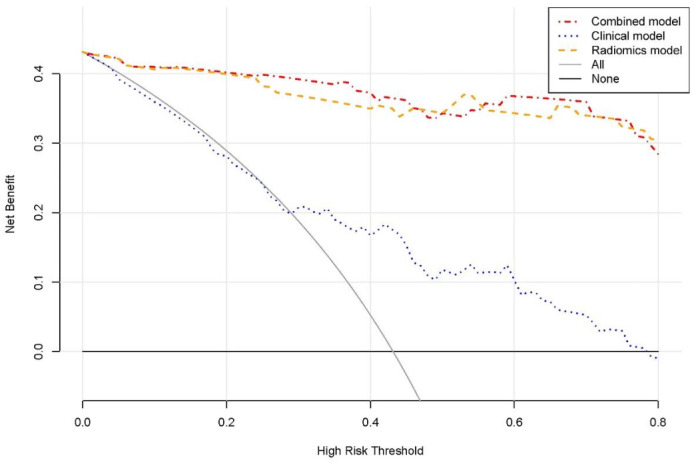
DCA for the combined model, clinical model and radiomics model in the training set.

The calibration curves of the combined model are depicted in Figure 6. It demonstrated that the combined model has a good agreement in predicting the MKI status in both the training and validation sets. The DCA results for the combined model, radiomics model and clinical model in the training and validation sets are shown in Figure 7. DCA shows that both the radiomics and combined models were added more net benefits than the clinical model in predicting the MKI status in neuroblastoma.

## 4. Discussion

MKI has been used to indirectly reflect the MYCN amplification [22], and it is independently prognostic in neuroblastoma [23]. Given the proven value of MKI status in the treatment and follow-up of neuroblastoma, MKI status is critical for risk stratification and prognostic prediction of neuroblastoma. Traditional MKI status analysis is invasive and may be hindered by factors such as potential tumor necrosis, patient refusal to suffer invasive testing, difficulties in the biopsy, and spatial and temporal heterogeneity of tumors, especially after chemotherapy. In addition, conventional MKI status is described by the number of mitotic and nucleated cells in multiple representative microscopic fields. This method has some limitations, such as the obvious difference between mitotic nuclei and karyorrhectic nuclei are sometimes obscure; the karyorrhectic cells, especially in the intermediate/high MKI cases, almost always exceed the mitotic cells in the same tumor tissues; the activities often vary greatly from area to area in intermediate MKI neuroblastoma [24]. However, the radiomics analysis of ^18^F-FDG PET/CT is expected to work out the above problems of MKI status in clinical practice. In this study, clinical characteristics and radiomics features were selected using a novel machine learning algorithm for the development of predictive models for the MKI status in pediatric neuroblastoma.

Radiomics can translate the spatial information of imaging voxels and changes in signal strength into higher dimensional information to quantify tumor heterogeneity and extract additional quantitative data that cannot be assessed by human eyes. In recent years, radiomics focuses on establishing the correlation between radiomics features and molecular biomarkers, is expected to supply an alternative, non-invasive, and inexpensive method for predicting various genetic tests for neuroblastoma.

Previous studies have reported radiomics potential role to predict molecular biomarkers in neuroblastoma, including MYCN in neuroblastoma by CT-based radiomics signature [8,25], tumor-associated macrophages by contrast-enhanced CT [26]. Moreover, some studies demonstrated that combining radiomics features with clinical characteristics can provide incremental predictive value for gene mutant status and expression. For example, it can be applied in the prediction of the epidermal growth factor receptor mutation status in lung adenocarcinoma [27] and MYCN amplification in neuroblastoma [28]. These previous radiomics researches on the prediction of gene mutations are based on single CT images only [29,30]. Compared with CT, ^18^F-FDG PET/CT can provide both anatomical and metabolic information in a single scan. Therefore, we built a novel radiomics model based on 13 radiomics features extracted from pre-therapy ^18^F-FDG PET/CT images via XGboost for predicting the MKI status. Among the 13 selected radiomics features in the present study, it demonstrated that features extracted from wavelet transformed images play an important role in prediction models. The wavelet transform can decompose the image into low-frequency elements and/or high-frequency components at different scales, and the texture features obtained from the wavelet decomposition of the original data can signify different frequency ranges within the tumor volume [31]. Some studies have demonstrated that wavelet-based features are important in radiomics studies and can show promising capabilities in terms of tumor classification and prognosis [32,33]. Our study also indicates the value of wavelet features in predicting MKI status.

In addition to radiomics analysis, we also evaluated the clinical characteristics. Finally, the age at diagnosis, serum LDH, urine HVA and VMA, and long tumor diameter were selected by XGboost to build the clinical model. The prognostic effects of MKI used in the INPC are age-dependent [23]. VMA and HVA levels in urine, the levels of serum LDH are considered characteristic tumor markers of neuroblastoma. These parameters are helpful at the initial diagnosis, response assessment, and monitoring recurrence of neuroblastoma [34]. A maximum primary tumor diameter greater than 13.20 cm is an independent risk factor for tumor rupture within high-risk neuroblastoma [35]. So, it was considered that MKI may be related to the above clinical characteristics, but in this study, when utilizing univariate analysis, there were no statistical differences in these clinical characteristics between the intermediate/high MKI group and the low MKI group, considering that this may be due to the small sample size. Moreover, the clinical model built with these clinical characteristics had an AUC of only 0.746 (training set) and 0.670 (validation set) in predicting MKI status.

In addition, we built a radiomics nomogram combining clinical characteristics and Rad-score for predicting the MKI status. Radiomics nomogram is an intuitive scoring system that can optimize the prediction efficacy of individuals by combining different variables. Our study demonstrated that the nomogram had a good performance in predicting the MKI status in pediatric neuroblastoma. Furthermore, the radiomics model showed a similar performance with radiomics nomogram. The study by Zhang et al. [36] also confirmed that both radiomics features and nomogram showed consistent predictive efficacy. Our results showed that both nomogram and radiomics models were better than the clinical model. The present study confirmed the potential value of radiomics based on ^18^F-FDG PET/CT in predicting the MKI status in pediatric neuroblastoma. This is one of the few radiomics-based studies focusing on MKI status in pediatric neuroblastoma.

The potential clinical value of our research is twofold: (1) it provides a relatively accurate, convenient, and noninvasive method for predicting MKI status in pediatric neuroblastoma patients; (2) the changes in radiomics features by PET and CT allow for a dynamic observation of MKI status before and after therapy.

Our study has several limitations. Firstly, the present study was a single-center design and included a relatively small sample size, which may influence the generalization ability of these models and affect their diagnostic efficacy. Moreover, this is a machine learning based study, and the small sample size may affect the robustness of the machine learning results, further amplifying the limitations of the sample size. Therefore, it is necessary to conduct multicenter studies in future studies to increase the sample size. Due to the small sample size, we lacked external validation in this research, However, a DCA was applied to assess the clinical usefulness of the combined, radiomics, and clinical models, demonstrating the great potential of the clinical utility of the radiomics for predicting MKI status. Secondly, all of the images were manually demarcated, which may lead to inconsistent and subjective tumor segmentation and degrade the performance of the model, so that further studies are needed to develop a uniform standard for multicenter studies and to establish and test multicenter imaging data by radiomics studies to make sure better robustness of the model. Furthermore, MKI status was divided into low and intermediate/high groups. In the future, MKI status was divided into three subgroups, low, intermediate, and high groups that may be more useful for clinical practice.

## 5. Conclusions

This study provides new comprehension into MKI status prediction in pediatric neuroblastoma. The above results suggest that the radiomics features can non-invasively predict the MKI status of pediatric neuroblastoma with high accuracy. It is a very effective tool for guiding the long-term management of pediatric neuroblastoma.

## Figures and Tables

**Table 1:** Characteristic of patients with neuroblastoma in the training set and validation set.

Characteristics	All Patients (*n* = 102)	Training Set (*n* = 68)	Validation Set (*n* = 34)	*p* Value
Age at diagnosis (months)	33.5 (17.0–52.3)	34.5 (16.3–51.8)	33.5 (19.8–64.5)	0.817
Sex				0.888
Female	55 (53.9)	37 (54.4)	18 (52.9)	
Male	47 (46.1)	31 (45.6)	16 (47.1)	
Long tumor diameter (cm)	9.4 (6.5–12.0)	10.2 (7.0–12.0)	7.6 (5.1–12.1)	0.094
INPC group				0.287
favorable	31 (30.4)	23 (33.8)	8 (23.5)	
unfavorable	71 (69.6)	45 (66.2)	26 (76.5)	
MYCN status				0.553
Amplified	15 (14.7)	9 (13.2)	6 (17.6)	
Not amplified	87 (85.3)	59 (86.8)	28 (82.4)	
INRG stage				0.447
L1, L2, MS	31	19 (27.9)	12 (35.3)	
M	71	49 (72.1)	22 (64.7)	
COG risk group				0.923
low	14 (13.7)	7 (10.3)	7 (20.6)	
intermediate	21 (20.6)	17 (25.0)	4 (11.8)	
high	67 (65.7)	44 (64.7)	23 (67.6)	
Mitosis-karyorrhexis index				0.572
Low	58 (56.9)	40 (58.8)	18 (52.9)	
Intermediate and high	44 (43.1)	28 (41.2)	16 (47.1)	
PET/CT findings				
SUVmax	4.8 (3.9–6.1)	4.7 (4.0–6.2)	4.9 (2.9–6.0)	0.580
SUVmean	2.0 (1.6–2.5)	2.0 (1.6–2.6)	1.9 (1.4–2.5)	0.482
MTV (mL)	167.7 (72.9–397.5)	192.9 (92.8–389.4)	126.9 (35.8–473.6)	0.194
TLG	348.5 (141.4–848.6)	391.8 (160.7–776.6)	206.3 (68.9–1028.8)	0.191
Initial laboratory findings				
NSE (ng/mL)	219.1 (65.4–626.3)	192.3 (69.3–531.1)	282.8 (47.9–686.6)	0.683
LDH (IU/L)	553.5 (341.8–1018.3)	495.0 (348.8–1046.8)	591.5 (339.3–998.3)	0.790
Ferritin (ng/mL)	118.3 (59.2–318.4)	117.2 (48.4–300.9)	150.9 (69.8–503.5)	0.407
HVA (μmol/L)	35.6 (11.0–107.2)	37.4 (13.8–111.9)	23.2 (3.9–103.4)	0.233
VMA (μmol/L)	149.5 (31.1–537.0)	188.1 (41.8–544.8)	106.8 (27.3–464.7)	0.268

INPC: International Neuroblastoma Pathology Classification; INRG: International Neuroblastoma Risk Group; COG: Children’s Oncology Group; NSE: Neuron specific enolase; LDH: Lactate dehydrogenase; HVA: Homovanillic acid; VMA: Vanillylmandelic acid.

**Table 2:** Characteristics of patients with neuroblastoma with low MKI and intermediate/high MKI.

Characteristics	Training Set			Validation Set		
	Low (*n* = 40)	Intermediate/High (*n* = 28)	*p* Value	Low (*n* = 18)	Intermediate/High (*n* = 16)	*p* Value
Age at diagnosis (months)	39.5 (16.3–52.8)	30 (16.3–47.8)	0.537	41 (20.8–69.0)	26.5 (16.0–38.5)	0.164
Sex			0.541			1.000
Female	23 (57.5)	14 (50.0)		10 (55.6)	8 (50.0)	
Male	17 (42.5)	14 (50.0)		8 (44.4)	8 (50.0)	
Long tumor diameter (cm)	8.5 (6.5–11.9)	10.6 (9.0–12.2)	0.079	6.7 (4.6–12.6)	9.9 (5.8–11.9)	0.325
INPC group			0.198			0.693
favorable	16 (40.0)	7 (25.0)		5 (27.8)	3 (18.8)	
unfavorable	24 (60.0)	21 (75.0)		13 (72.2)	13 (81.3)	
MYCN status			0.192			0.387
Amplified	3 (7.5)	6 (21.4)		2 (11.1)	4 (25.0)	
Not amplified	37 (92.5)	22 (78.6)		16 (88.9)	12 (75.0)	
INRG stage			0.651			0.729
L1, L2, MS	12 (30.0)	7 (25.0)		7 (38.9)	5 (31.3)	
M	28 (70.0)	21 (75.0)		11 (61.1)	11 (68.8)	
COG risk group			0.707			0.443
low	4 (10.0)	3 (10.7)		5 (27.8)	2 (12.5)	
intermediate	11 (27.5)	6 (21.4)		2 (11.1)	2 (12.5)	
high	25 (62.5)	19 (67.9)		11 (61.1)	12 (75.0)	
PET/CT findings						
SUVmax	4.4 (4.0–5.8)	5.0 (4.1–6.7)	0.174	4.3 (2.4–5.9)	5.5 (4.1–6.2)	0.102
SUVmean	1.9 (1.6–2.4)	2.2 (1.7–2.7)	0.148	1.7 (1.3–2.3)	2.3 (1.6–2.9)	0.050
MTV (mL)	185.9 (80.8–389.4)	218.3 (127.8–396.1)	0.360	68.5 (8.6–257.4)	239.7 (80.8–565.9)	0.088
TLG	369.9 (141.2–644.7)	567.1 (211.9–1054.1)	0.204	138.6 (12.0–640.8)	593.9 (157.3–1463.4)	0.039
Initial laboratory findings						
NSE (ng/mL)	177.7 (60.7–340.6)	260.2 (91.3–747.9)	0.195	143.1 (28.3–552.3)	459.0 (141.7–736.9)	0.164
LDH (IU/L)	485 (357.3–738.8)	762 (329.0–1418.5)	0.189	545.0 (285.0–896.5)	659.0 (358.0–1019.0)	0.246
Ferritin (ng/mL)	111.8 (42.8–269.1)	138.1 (65.2–358.5)	0.294	302.3 (42.1–687.9)	117.1 (90.3–210.2)	0.297
HVA (μmol/L)	39.5 (22.3–101.9)	31.6 (8.7–152.6)	0.451	63.8 (3.4–153.8)	20.5 (4.0–47.5)	0.589
VMA (μmol/L)	255.3 (79.3–587.3)	59.2 (23.9–375.7)	0.052	195.2 (27.2–614.0)	48.1 (27.7–290.0)	0.650

MKI: Mitosis-karyorrhexis index; INPC: International Neuroblastoma Pathology Classification; INRG: International Neuroblastoma Risk Group; COG: Children’s Oncology Group; NSE: Neuron specific enolase; LDH: Lactate dehydrogenase; HVA: Homovanillic acid; VMA: Vanillylmandelic acid.

**Table 3:** Predictive performance of three models in the training and validation sets.

Model	Training Set		Validation Set	
	AUC (95%CI)	*p*	AUC (95%CI)	*p*
radiomics model	0.982 (0.916–0.999)		0.955 (0.823–0.997)	
clinical model	0.746 (0.625–0.843)		0.670 (0.488–0.821)	
combined model	0.988 (0.924–1.000)		0.951 (0.818–0.996)	
radiomics model vs clinical model		0.0001		0.0086
radiomics model vs combined model		0.2625		0.8807
clinical model vs combined model		<0.0001		0.0046

AUC: Area under the curve; CI: Confidence interval.

## Data Availability

The data presented in this study are available on request from the corresponding author.
